# Food Sources of Energy and Nutrients among Children in the United States: National Health and Nutrition Examination Survey 2003–2006

**DOI:** 10.3390/nu5010283

**Published:** 2013-01-22

**Authors:** Debra R. Keast, Victor L. Fulgoni III, Theresa A. Nicklas, Carol E. O’Neil

**Affiliations:** 1 Food & Nutrition Database Research, Inc., 1801 Shadywood Lane, Okemos, MI 48864, USA; E-Mail: keastdeb@comcast.net; 2 Nutrition Impact, LLC, 9725 D Drive North, Battle Creek, MI 49014, USA; E-Mail: vic3rd@aol.com; 3 Children’s Nutrition Research Center, Baylor College, Department of Pediatrics, 1100 Bates Avenue, Houston, TX 77030, USA; E-Mail: tnicklas@bcm.tmc.edu; 4 Didactic Program in Dietetics, 261 Knapp Hall, Louisiana State University AgCenter, Baton Rouge, LA 70803, USA

**Keywords:** NHANES, energy intake, nutrients, children, adolescents, food groups

## Abstract

Background: Recent detailed analyses of data on dietary sources of energy and nutrients in US children are lacking. The objective of this study was to identify food sources of energy and 28 nutrients for children in the United States. Methods: Analyses of food sources were conducted using a single 24-h recall collected from children 2 to 18 years old (*n* = 7332) in the 2003–2006 National Health and Nutrition Examination Survey. Sources of nutrients contained in foods were determined using nutrient composition databases. Food grouping included ingredients from disaggregated mixtures. Mean energy and nutrient intakes from the total diet and from each food group were adjusted for the sample design using appropriate weights. Percentages of the total dietary intake that food sources contributed were tabulated by rank order. Results: The two top ranked food/food group sources of energy and nutrients were: energy—milk (7% of energy) and cake/cookies/quick bread/pastry/pie (7%); protein—milk (13.2%) and poultry (12.8%); total carbohydrate—soft drinks/soda (10.5%) and yeast bread/rolls (9.1%); total sugars—soft drinks/soda (19.2%) and yeast breads and rolls (12.7%); added sugars—soft drinks/soda (29.7%) and candy/sugar/sugary foods (18.6%); dietary fiber—fruit (10.4%) and yeast bread/rolls (10.3%); total fat—cheese (9.3%) and crackers/popcorn/pretzels/chips (8.4%); saturated fatty acids—cheese (16.3%) and milk (13.3%); cholesterol—eggs (24.2%) and poultry (13.2%); vitamin D—milk (60.4%) and milk drinks (8.3%); calcium—milk (33.2%) and cheese (19.4%); potassium—milk (18.8%) and fruit juice (8.0%); and sodium—salt (18.5%) and yeast bread and rolls (8.4%). Conclusions: Results suggest that many foods/food groupings consumed by children were energy dense, nutrient poor. Awareness of dietary sources of energy and nutrients can help health professionals design effective strategies to reduce energy consumption and increase the nutrient density of children’s diets.

## Abbreviations

CSFII: Continuing Study of Food Intake by Individuals
DSN: Dietary Sources of Nutrients
FNDDS: Food and Nutrient Database for Dietary Studies
kcals: kilocalories
MUFA: mono-unsaturated fatty acids
NHANES: National Health and Nutrition Examination Survey
PUFA: poly-unsaturated fatty acids
RTEC: ready-to-eat cereal
SFA: saturated fatty acids
SR: Standard Reference
USDA: United States Department of Agriculture

## 1. Introduction

The US food supply provides an overabundance of energy and nutrients [1]; however, most children do not consume the amounts and types of foods consistent with dietary recommendations [2,3]. Children, especially young children, have high nutrient needs for growth and development, but relatively modest energy requirements, leaving little room for energy-dense, nutrient-poor foods in the diet [4]. Thus, it is important to encourage nutrient dense food choices and diets. Dietary preferences are established early in life [5,6,7]. Establishing a healthful diet in childhood may help reduce the risk of developing diet-related chronic diseases in adulthood [8,9,10]. 

Critical nutrition concerns for US children include excessive intake of energy which may increase the risk for weight gain and development of obesity. Data from the 2007–2008 National Health and Nutrition Examination Survey (NHANES) showed that among children 2 to 19 years (y), 31.7% were overweight and 16.9% were obese [11]. Overweight/obesity in children has been associated with adverse lipid levels, blood glucose derangements, elevated blood pressure [12,13,14], behavioral and psychosocial problems [15,16], and adult atherosclerosis [17] and obesity [12,13,14,18]. Excess consumption of saturated fatty acids (SFA) has been associated with increased plasma total and low-density lipoprotein cholesterol in childhood [19,20,21], which could increase cardiovascular disease risk; however, recent research has brought this association into question [22,23]. Inadequate consumption of foods rich in dietary fiber, vitamin D, calcium, and potassium [24,25] are major public health concerns. Intake of dietary fiber has been associated with decreased heart disease, obesity, and type 2 diabetes risks [26]. Inadequate intake of nutrients, such as vitamin D and calcium, in combination with a sedentary lifestyle in childhood, can impede the achievement of maximal bone mineral content and density, thereby increasing the diet-related risk of developing osteoporosis later in life [27]. In childhood, overconsumption of sodium, especially with inadequate intake of potassium, has been associated with hypertension [28].

Recent detailed analyses of data on dietary sources of energy and nutrients in US children are lacking. In 1998, Subar *et al.* [29] reported disaggregated dietary sources of energy and nutrients among US children from the 1989 to 1991 Continuing Survey of Food Intakes by Individuals (CSFII). Recently, using data from the 2003–2004 or 2005–2006 NHANES, dietary sources of total energy and energy from solid fats and added sugars, respectively, were reported [30]. The purpose of this study was to update previous research by examining dietary sources of energy and 28 nutrients using data from a recent nationally representative sample of US children 2 to 18 y. Understanding what children are consuming can help shape appropriate interventions to improve their diet and ultimately their health.

## 2. Methods

### 2.1. Population and Dietary Intake

The survey design, questionnaires, and examination methodology of NHANES have been described previously [31]. Intake data from children 2 to 18 y (*n* = 7332) participating in the 2003–2006 NHANES were obtained from in-person 24-h dietary recall interviews using an automated multiple-pass method [32,33]. Descriptions of dietary interview methods have also been described previously [34]. Data judged incomplete or unreliable by the Food Surveys Research Group were excluded from analyses. Since this was a secondary data analysis, the current study was exempted by the Louisiana State University Agricultural Center Institutional Review Board.

### 2.2. Food Groupings and Composition

The USDA Dietary Sources of Nutrients (DSN) database [35] was used to define food groups. The DSN database was originally developed for use with the CSFII 1994–1996. Therefore, for this research the DSN database was updated for application to recent food consumption surveys. The food grouping and disaggregation rules used to update the DSN database were similar to those methods reported by others who have described dietary sources of nutrients in the American diet [36,37]. The more than 130 DSN food groups were collapsed into 51 categories, an aggregation level consistent with that used by the USDA Food Surveys Research Group when defining food groups [38,39]. 

If foods were not disaggregated in the DSN database, the Food and Nutrient Database for Dietary Studies (FNDDS) codes were assigned to DSN food groups (FNDDS versions 2.0 [38] and 3.0 [39] were used in 2003–2004 and 2005–2006 NHANES, respectively). The ingredients of disaggregated survey food recipes (coded using the USDA Nutrient Database for Standard Reference [SR] food codes) were linked to the appropriate food composition databases using the SR-Link file of the FNDDS (versions 2.0 [38] and 3.0 [39] link SR releases 18 [40] and 20 [41], respectively). Recipe calculations were performed to determine proportions of the disaggregated survey foods assigned to the 51 DSN food groups.

### 2.3. Statistical Analyses

Analyses were conducted using SUDAAN 9.0.3 (Research Triangle Institute, Research Triangle Park, NC, USA; 2007). Appropriate weighting factors were used in analyses to adjust for oversampling of selected groups, survey non-response of some individuals, and day of the week the interview was conducted [42]. Mean and standard errors of energy and nutrient intakes from the total diet and from each food group were determined using PROC DESCRIPT of SUDAAN. Using the mean intake data, the average percentage of total dietary intake of energy and nutrients contributed from each food group was calculated and tabulated in ranked order.

## 3. Results

Dietary sources of energy and 28 nutrients are shown in Table 1, Table 2, Table 3, Table 4, Table 5, Table 6, Table 7, Table 8, Table 9, Table 10, Table 11, Table 12 and Table 13 (energy, macronutrients—protein, carbohydrates and total fat, nutrients to decrease—saturated fat, cholesterol, total sugars, added sugars and sodium and nutrients to increase—dietary fiber, vitamin D, calcium and potassium) based on US dietary Guidelines [25] in the text and in Supplemental Tables S1–S16, which are available online. 

### 3.1. Energy, Macronutrients, Cholesterol and Dietary Fiber

Mean dietary sources of energy, protein, carbohydrate, total and added sugars, dietary fiber, total fat, SFA, and cholesterol are presented in Table 1, Table 2, Table 3, Table 4, Table 5, Table 6, Table 7, Table 8 and Table 9, respectively. More than 20 food groups contributed to energy. The two highest ranked dietary sources, milk (whole, reduced fat, and fat-free) and cake/cookies/quick bread/pastry/pie, each provided 7% of total energy intake. Other dietary sources providing at least 5% of energy intake were yeast breads/rolls (6.7%), crackers/popcorn/pretzels/chips (6.0%); soft drinks/soda (5.5%) and candy/sugars/sugary foods (5.1%) (Table 1). 

Milk (13.2%) was the highest ranked dietary source of protein in children’s diets followed by poultry (12.8%); beef (11.5%); cheese (9.7%); and yeast breads/rolls (6.4%) (Table 2). Soft drinks/soda (10.5%) was the highest ranked food group source of carbohydrate, followed by yeast breads/rolls (9.1%), candy/sugars/sugary foods (8.3%), cake/cookies/quick bread/pastry/pie (7.9%), and fruit drinks/ades (6.6%) (Table 3). The top sources of total sugars were soft drinks/soda (19.2%) and candy/sugars/sugary food (12.7%) (Table 4); the top sources of added sugars were soft drinks/soda (29.7%) and candy/sugars/sugary food (18.6%) (Table 5). The five highest ranked dietary sources of dietary fiber were fruit (10.4%), yeast breads/rolls (10.3%), crackers/popcorn/pretzels/chips (8.3%), potatoes (white) (8.3%), and ready-to-eat cereal (RTEC) (6.3%) (Table 6). The highest ranking dietary sources of total fat were cheese (9.3%) and crackers/popcorn/pretzels/chips (8.4%) (Table 7); the three highest ranked dietary sources of SFA were cheese (16.3%), milk (13.3%) and frankfurters/sausages/luncheon meat (7.4%) (Table 8). Eggs (24.2%) and poultry (13.3%) were the highest ranking sources of cholesterol (Table 9). 

**Table 1:** Food/food group sources of energy among US children aged 2–18 years (from NHANES 2003–2006) ^a^.

Food/Food Group ^b^	Energy (mean = 2072 kcal)		
	Ranking	% Total	Cumulative %
Milk	1	7.0	7.0
Cake, cookies, quick bread, pastry, pie	2	7.0	14.0
Yeast breads and rolls	3	6.7	20.7
Crackers, popcorn, pretzels, chips	4	6.0	26.7
Soft drinks, soda (includes diet)	5	5.5	32.2
Candy, sugars and sugary foods	6	5.1	37.3
Cheese	7	4.7	42.0
Poultry	8	4.4	46.4
Beef	9	3.8	50.2
Fruit drinks, and ades	10	3.5	53.7
Ready-to-eat cereal	11	3.4	57.1
Biscuits, corn bread, pancakes, tortillas	12	3.4	60.5
Frankfurters, sausages, luncheon meats	13	3.2	63.7
Potatoes (white)	14	3.1	66.8
Flour, bran, baking ingredients	15	2.9	69.7
Milk desserts	16	2.8	72.5
Fruit juices	17	2.7	75.2
Mixtures, mostly grain	18	2.7	77.9
Other fats and oils	19	2.6	80.5
Pasta	20	2.2	82.7
Fruit	21	2.0	84.7

^a^ Data are for children aged 2 to 18 years (*n* = 7332), Day 1 intakes; ^b^ Food groups (*n* = 6) contributing at least 1% in descending order: milk drinks; salad dressings, mayonnaise; margarine and butter; nuts, seeds (including butters, pastes); pork, ham, bacon; and rice, cooked grains.

**Table 2:** Food/Food group sources of protein among US children aged 2–18 years (from NHANES 2003–2006) ^a^.

Food/Food Group ^b^	Protein (mean = 71.6 g)		
	Ranking	% Total	Cumulative %
Milk	1	13.2	13.2
Poultry	2	12.8	26.0
Beef	3	11.5	37.5
Cheese	4	9.7	47.2
Yeast breads and rolls	5	6.4	53.6
Frankfurters, sausages, luncheon meats	6	4.8	58.4
Pork, ham, bacon	7	4.2	62.6
Mixtures, mostly grain	8	3.5	66.1
Crackers, popcorn, pretzels, chips	9	2.6	68.7
Cakes, cookies, quick bread, pastry, pie	10	2.6	71.3
Flour, bran, baking ingredients	11	2.4	73.7
Pasta	12	2.4	76.1
Biscuits, corn bread, pancakes, tortillas	13	2.4	78.5
Eggs	14	2.3	80.8
Fish and shellfish	15	2.1	82.9

^a^ Data are for children aged 2 to 18 years (*n* = 7332), Day 1 intakes; ^b^ Food groups (*n* = 6) contributing at least 1% in descending order: milk drinks; ready-to-eat cereal; nuts, seeds (including butters, pastes); milk desserts; potatoes (white); and legumes.

**Table 3:** Food/Food group sources of carbohydrate among US children aged 2–18 years (from NHANES 2003–2006) ^a^.

Food/Food Group ^b^	Carbohydrate (mean = 278 g)		
	Ranking	% Total	Cumulative %
Soft drinks, soda	1	10.5	10.5
Yeast breads and rolls	2	9.1	19.6
Candy, sugars and sugary foods	3	8.3	27.9
Cake, cookies, quick bread, pastry, pie	4	7.9	35.8
Fruit drinks and ades	5	6.6	42.4
Ready-to-eat cereal	6	5.6	48.0
Crackers, popcorn, pretzels, chips	7	5.5	53.5
Fruit juices	8	5.0	58.5
Milk	9	4.8	63.3
Flour, bran, baking ingredients	10	4.6	67.9
Biscuits, corn bread, pancakes, tortillas	11	3.9	71.8
Fruit	12	3.9	75.7
Potatoes (white)	13	3.4	79.1
Pasta	14	3.3	82.4
Milk desserts	15	2.8	85.2
Mixtures, mostly grain	16	2.1	87.3
Milk drinks	17	2.1	89.4

^a^ Data are for children aged 2 to 18 years (*n* = 7332), Day 1 intakes; ^b^ Food groups (*n* = 2) contributing at least 1% in descending order: rice, cooked grains and tomatoes, tomato/vegetable juice.

**Table 4:** Food/Food group sources of total sugars among US children aged 2–18 years (from NHANES 2003–2006) ^a^.

Food/Food Group ^b^	Total Sugars (mean = 142 g)		
	Ranking	% Total	Cumulative %
Soft drinks, soda	1	19.2	19.2
Candy, sugars and sugary foods	2	12.7	31.9
Fruit drinks and ades	3	11.4	43.3
Milk	4	10.4	53.7
Fruit juice	5	8.8	62.5
Cake, cookies, quick bread, pastry, pie	6	8.6	71.1
Fruit	7	5.5	76.6
Milk desserts	8	4.5	81.1
Ready-to-eat cereals	9	4.2	85.3
Milk drinks	10	3.4	88.7

^a^ Data are for children aged 2 to 18 years (*n* = 7332), Day 1 intakes; ^b^ Food groups (*n* = 4) contributing at least 1% in descending order: yeast breads and rolls; yogurt; tomatoes, tomato/vegetable juice; and condiments and sauces.

**Table 5:** Food/Food group sources of added sugars among US children aged 2–18 years (from NHANES 2003–2006) ^a^.

Food/Food Group ^b^	Added Sugars (mean = 91.9 g)		
	Ranking	% Total	Cumulative %
Soft drinks, soda (includes diet)	1	29.7	29.7
Candy, sugars and sugary foods	2	18.6	48.3
Fruit drinks and ades	3	15.4	63.7
Cake, cookies, quick bread, pastry, pie	4	12.2	75.9
Ready-to-eat cereal	5	6.3	82.2
Milk desserts	6	5.7	87.9
Milk drinks	7	3.2	91.1
Yeast breads and rolls	8	2.3	93.4

^a^ Data are for children aged 2 to 18 years (*n* = 7332), Day 1 intakes; ^b^ Food groups (*n* = 2) contributing at least 1% in descending order: yogurt and condiments and sauces.

**Table 6:** Food/Food group sources of dietary fiber among US children aged 2–18 years (from NHANES 2003–2006) ^a^.

Food/Food Group ^b^	Dietary Fiber (mean = 12.8 g)		
	Ranking	% Total	Cumulative %
Fruit	1	10.4	10.4
Yeast breads and rolls	2	10.3	20.7
Crackers, popcorn, pretzels, chips	3	8.3	29.0
Potatoes (white)	4	6.9	35.9
Ready-to-eat cereal	5	6.3	42.2
Biscuits, corn bread, pancakes, tortillas	6	5.7	47.9
Legumes	7	5.5	53.4
Cake, cookies, quick bread, pastry, pie	8	5.1	58.5
Pasta	9	4.1	62.6
Tomatoes, tomato/vegetable juice	10	4.0	66.6
Mixtures, mostly grain	11	4.0	70.6
Flour, bran, baking ingredients	12	4.0	74.6
Nuts, seeds (including butters, pastes)	13	2.8	77.4
Milk drinks	14	2.7	80.1
Other vegetables	15	2.6	82.7

^a^ Data are for children aged 2 to 18 years (*n* = 7332), Day 1 intakes; ^b^ Food groups (*n* = 8) contributing at least 1% in descending order: candy, sugars and sugary foods; corn, peas, lima beans; milk desserts; fruit juice; poultry; carrots, sweet potatoes, winter squash; broccoli, spinach, greens; and condiments and sauces.

**Table 7:** Food/Food group sources of total fat among US children aged 2–18 years (from NHANES 2003–2006) ^a^.

Food/Food Group ^b^	Total Fat (mean = 77.0 g)		
	Ranking	% Total	Cumulative %
Cheese	1	9.3	9.3
Crackers, popcorn, pretzels, chips	2	8.4	17.7
Milk	3	7.7	25.4
Other fats and oils	4	7.5	32.9
Cake, cookies, quick bread, pastry, pie	5	7.5	40.4
Frankfurters, sausages, luncheon meats	6	7.2	47.6
Poultry	7	6.2	53.8
Beef	8	6.2	60.0
Salad dressings, mayonnaise	9	4.7	64.7
Margarine and butter	10	4.4	69.1
Potatoes (white)	11	3.5	72.6
Milk desserts	12	3.4	76.0
Mixtures, mostly grain	13	3.2	79.2
Nuts, seeds (including butters, pastes)	14	3.1	82.3
Biscuits, corn bread, pancakes, tortillas	15	2.9	85.2
Yeast breads and rolls	16	2.6	87.8
Pork, ham, bacon	17	2.2	90.0
Candy, sugars and sugary foods	18	2.1	92.1

^a^ Data are for children aged 2 to 18 years (*n* = 7332), Day 1 intakes; ^b^ Food groups (*n* = 2) contributing at least 1% in descending order: eggs and milk drinks.

**Table 8:** Food/Food group sources of saturated fatty acids among US children aged 2–18 years (from NHANES 2003–2006) ^a^.

Food/Food Group ^b^	Saturated Fatty Acids (mean = 27.2 g)		
	Ranking	% Total	Cumulative %
Cheese	1	16.3	16.3
Milk	2	13.3	29.6
Frankfurters, sausages, luncheon meats	3	7.4	37.0
Beef	4	6.7	43.7
Other fats and oils	5	5.8	49.5
Milk desserts	6	5.8	55.3
Cake, cookies, quick bread, pastry, pie	7	5.8	61.1
Crackers, popcorn, pretzels, chips	8	5.0	66.1
Poultry	9	4.3	70.4
Margarine and butter	10	4.2	74.6
Mixtures, mostly grain	11	3.7	78.3
Candy, sugars, and sugary foods	12	2.8	81.1
Potatoes (white)	13	2.3	83.4
Milk drinks	14	2.2	85.6
Pork, ham, bacon	15	2.1	87.7
Salad dressings, mayonnaise	16	2.0	89.7

^a^ Data are for children aged 2 to 18 years (*n* = 7332), Day 1 intakes; ^b^ Food groups (*n* = 4) contributing at least 1% in descending order: biscuits, corn bread, pancakes, tortillas; yeast breads and rolls; nuts, seeds (including butters, pastes); and eggs.

**Table 9:** Food/Food group sources of cholesterol among US children aged 2–18 years (from NHANES 2003–2006) ^a^.

Food/Food Group ^b^	Cholesterol (mean = 267 mg)		
	Ranking	% Total	Cumulative %
Eggs	1	24.2	24.2
Poultry	2	13.2	37.4
Beef	3	11.5	48.9
Milk	4	9.7	58.6
Cheese	5	9.6	68.2
Frankfurters, sausages, luncheon meats	6	6.6	74.8
Milk desserts	7	4.1	78.9
Pork, ham, bacon	8	3.8	82.7
Cake, cookies, quick bread, pastry, pie	9	3.4	86.1
Mixtures, mostly grain	10	2.6	88.7
Fish and shellfish	11	2.1	90.8

^a^ Data are for children aged 2 to 18 years (*n* = 7332), Day 1 intakes; ^b^ Food groups (*n* = 4) contributing at least 1% in descending order: biscuits, corn bread, pancakes, tortillas; milk drinks; margarine and butter; other fats and oils.

### 3.2. Micronutrients

Sources of vitamin D, calcium, and potassium, are shown in Table 10, Table 11 and Table 12, respectively. Milk was the highest ranked dietary source of calcium (33.2%), vitamin D (60.4%), and potassium (18.8%) (Table 10, Table 11 and Table 12). Other dietary sources of vitamin D food included milk drinks (8.3%), RTEC (8.2%), fish/shellfish (3.5%), and fruit juice (3.0%) (Table 10). Other major dietary sources of calcium included cheese (19.4%), yeast breads/rolls (6.0%), milk drinks (5.0%), and milk desserts (3.7%) (Table 11). Other dietary sources of potassium included fruit juice (8.0%), potatoes (6.7%), fruit (5.4%), and crackers/popcorn/pretzels/chips (5.3%) (Table 12). The highest ranked dietary source of sodium was salt separated from other ingredients of disaggregated foods (18.5%) followed by yeast breads/rolls (8.4%), cheese (8.1%), frankfurters/sausages/luncheon meats (7.4%), and crackers/popcorn/pretzels/chips (6.1%) (Table 13).

**Table 10:** Food/Food group sources of vitamin D among US children aged 2–18 years (from NHANES 2003–2006) ^a^.

Food/Food Group ^b^	Vitamin D (mean = 5.8 μg)		
	Ranking	% Total	Cumulative %
Milk	1	60.4	60.4
Milk drinks	2	8.3	68.7
Ready-to-eat cereal	3	8.2	76.9
Fish and shellfish	4	3.5	80.4
Fruit juice	5	3.0	83.4
Eggs	6	2.7	86.1
Frankfurters, sausages, luncheon meats	7	2.4	88.5
Cheese	8	2.3	90.8

^a^ Data are for children aged 2 to 18 years (*n* = 7332), Day 1 intakes; ^b^ Food groups (*n* = 4) contributing at least 1% in descending order: pork, ham, bacon; margarine and butter; yogurt; and beef.

**Table 11:** Food/Food group sources of calcium among US children aged 2–18 years (from NHANES 2003–2006) ^a^.

Food/Food Group ^b^	Calcium (mean = 1101 mg)		
	Ranking	% Total	Cumulative %
Milk	1	33.2	33.2
Cheese	2	19.4	52.6
Yeast breads and rolls	3	6.0	58.6
Milk drinks	4	5.0	63.6
Milk desserts	5	3.7	67.3
Biscuits, corn bread, pancakes, tortillas	6	3.2	70.5
Fruit juice	7	3.2	73.7
Mixtures, mostly grain	8	3.2	76.9
Ready-to-eat cereal	9	2.8	79.7
Cake, cookies, quick bread, pastry, pie	10	2.0	81.7
Crackers, popcorn, pretzels, chips	11	2.0	83.7

^a^ Data are for children aged 2 to 18 years (*n* = 7332), Day 1 intakes; ^b^ Food groups (*n* = 3) contributing at least 1% in descending order: coffee, tea, other, nonalcoholic beverages; yogurt; and fruit drinks and ades.

**Table 12:** Food/Food group sources of potassium among US children aged 2–18 years (from NHANES 2003–2006) ^a^.

Food/Food Group ^b^	Potassium (mean = 2266 mg)		
	Ranking	% Total	Cumulative %
Milk	1	18.8	18.8
Fruit juice	2	8.0	26.8
Potatoes (white)	3	6.7	33.5
Fruit	4	5.4	38.9
Crackers, popcorn, pretzels, chips	5	5.3	44.2
Tomatoes, tomato/vegetable juice	6	5.1	49.3
Beef	7	4.4	53.7
Poultry	8	4.1	57.8
Milk drinks	9	3.2	61.0
Milk desserts	10	2.7	63.7
Yeast breads and rolls	11	2.6	66.3
Frankfurters, sausages, luncheon meats	12	2.4	68.7
Cake, cookies, quick bread, pastry, pie	13	2.1	70.8
Cheese	14	2.0	72.8
Pork, ham, bacon	15	2.0	74.8

^a^ Data are for children aged 2 to 18 years (*n* = 7332), Day 1 intakes; ^b^ Food groups (*n* = 11) contributing at least 1% in descending order: condiments and sauces; legumes; ready-to-eat cereal; mixtures, mostly grain; other vegetables; biscuits, corn bread, pancakes, tortillas; fruit drinks and ades; nuts, seeds (including butters, pastes); candy, sugars and sugary foods; flour, bran, baking ingredients; and coffee, tea, other, nonalcoholic beverages.

**Table 13:** Food/Food group sources of sodium among US children aged 2–18 years (from NHANES 2003–2006) ^a^.

Food/Food Group ^b^	Sodium (mean = 3158 mg)		
	Ranking	% Total	Cumulative %
Salt	1	18.5	18.5
Yeast breads and rolls	2	8.4	26.9
Cheese	3	8.1	35.0
Frankfurters, sausages, luncheon meats	4	7.4	42.4
Crackers, popcorn, pretzels, chips	5	6.1	48.5
Condiments and sauces	6	5.4	53.9
Biscuits, corn bread, pancakes, tortillas	7	4.2	58.1
Pork, ham, bacon	8	3.8	61.9
Milk	9	3.8	65.7
Mixtures, mostly grain	10	3.7	69.4
Cake, cookies, quick bread, pastry, pie	11	3.5	72.9
Ready-to-eat cereal	12	3.5	76.4
Poultry	13	3.3	79.7
Soup, broth, bouillon	14	2.8	82.5
Tomatoes, tomato/vegetable juice	15	2.4	84.9

^a^ Data are for children aged 2 to 18 years (*n* = 7332), Day 1 intakes; ^b^ Food groups (*n* = 5) contributing at least 1% in descending order: potatoes (white); margarine and butter; salad dressings, mayonnaise; olives, pickles; fruit drinks and ades.

## 4. Discussion

The detailed analysis of children’s nutrient intake by Subar *et al.* [37] used data from the 1989–1991 CSFII that is now more than two decades old. Since then, forces driving trends in children’s food/beverage consumption have included an increased number of meals consumed outside the home [43]; trade liberalization which has increased the number of foods available [44]; changing beverage [45,46], food [47], and snacking preferences [48]; availability of competitive foods in schools [49]; and food product reformulations in response to consumers’ health concerns [50]. More recent data [30] only provided information on total energy, solid fat, and added sugar intake among children. Thus, it was important to provide a more detailed analysis of children’s diet using recent nationally representative data. Faced with a pediatric obesity epidemic, it is important to identify principal sources of energy. This study showed that major sources of energy in children’s diets are not necessarily the same food groups that provide rich sources of nutrients. Of the 10 highest ranked foods/food groups, five groups including milk, yeast bread/rolls, cheese, poultry, and beef accounted for approximately 27% of energy intake; these foods/food groups provided a variety of nutrients, including calcium, vitamin D, and potassium. In their lowest fat form, these foods/food groups are recommended by MyPlate [51]. In contrast the other five food/food groups that were major energy sources (27% combined): cake/cookies/quick bread/pastry/pie, crackers/popcorn/pretzels/chips, soft drinks/soda, fruit drinks/ades, and candy/sugars/sugary foods, contributed mainly fat or added sugars and few other nutrients. That many of the major sources of energy are nutrient-poor foods is consistent with reports that many children do not follow dietary recommendations, and without consuming recommended amounts of nutrient-dense foods, such as whole grains, fruit, vegetables, low-fat milk, and lean meat, diets can be nutritionally inadequate [3]. 

Soft drinks/soda and fruit drinks/ades contributed 5.5% and 3.5% of energy, respectively; if these two sweetened beverage categories were combined, they provided a higher percentage (9%) than any other source of energy in the diet. Subar *et al.* [29] also showed that soft drinks and soda were major contributors of energy to the diets of children (4.3%); however, in that study, fruit drinks provided only 2.2% of energy intake. Reedy, *et al.* [30] also reported soft drinks and soda as a major source of energy. From 1977 to 2006, children’s beverage consumption patterns changed and intake of sweetened beverages increased from 87 to 154 kcals [45]. 

Subar *et al.* [29] reported that the five highest ranked energy sources were milk (11.7%), yeast bread (9.3%), cakes/cookies/quick breads/donuts (6.2%), beef (5.7%), RTEC (4.5%), and soft drinks/soda (4.3%). A comparison of that study and ours suggests a temporal shift in sources of energy occurred. Since our study combined crackers/pretzels with popcorn/chips, and candy with sugars/sugary foods, the ranking of the combined groups was higher than each of the individual groups studied in Subar’s study.

Data from children in the 2005–2006 NHANES [30] showed the five highest ranked sources of energy were grain desserts (e.g., cakes) (6.8% of total energy consumed), pizza (6.7%), soda (5.8%), yeast breads (5.6%), and chicken (5.6%). In that study, mixtures were not disaggregated. Grain desserts and soda were identified in that study and ours as major contributors of energy to the diets of children. The study using 2005–2006 NHANES data separated milk by fat level, and therefore milk was not identified as a major source of energy in children’s diets; however, if whole and reduced-fat milk were combined they contributed the highest percentage of energy to the diet.

The same five highest sources of protein were identified by Subar *et al.* [29], albeit with a different rank order. Cheese and milk were the two highest ranked sources of SFA, followed by frankfurters/sausages/luncheon meats. The contribution of SFA in the diet by milk, beef and frankfurters/sausages/luncheon meats shown in our study was lower and cheese and crackers/popcorn/pretzels/chips was higher when compared with the 1989–1992 CSFII data [29]. Current dietary recommendations are to replace SFA with mono-unsaturated fatty acids (MUFA) and poly-unsaturated fatty acids (PUFA) [25] since the latter are associated with decreased cardiovascular risk [52]. Our study showed that of the five highest ranked sources of MUFA (cake/cookies/quick bread/pastry/pie, frankfurters/sausages/luncheon meats, other fats/oils, beef, and crackers/popcorn/pretzels/chips) (Supplemental Tables); beef was the only nutrient dense food. Of the five highest ranked sources of PUFA, poultry was the only nutrient-dense food. Intake of nutrient dense sources of oils, such as nuts, should be encouraged (Supplemental Tables).

The 2010 Dietary Guidelines for Americans recognized the low intakes of dietary fiber, calcium, vitamin D and potassium were of public health concern [25]. Ideally, diets or food patterns containing adequate amounts of these nutrients should be recommended so that intake of nutrient-dense foods can be consumed without unduly increasing energy intake. In children, dietary fiber intake is inversely associated with serum cholesterol levels [53] and constipation [54], a major cause of morbidity in children [55]. Dietary fiber intake by children is approximately half of what is recommended [56]. Fruit was the highest ranked source; however, most children do not consume the recommended amount of fruit [3]. Fruit, yeast bread/rolls, crackers/popcorn/pretzels/chips, potatoes, RTEC, biscuits/corn bread/pancakes/tortillas, legumes, and cake/cookies/quick bread/pastry/pie provided a cumulative 58.5% of fiber intake. Of these foods/food groups, fruit, yeast bread/rolls, potatoes, RTEC, and legumes provide other nutrients while most of the other food/food groups do not. Cake/cookies/quick bread/pastry/pie provided both fat and added sugars and were poor sources of most other nutrients. Because vegetables were split into many different categories, vegetables, other than potatoes, each contributed 4% or less of dietary fiber; however, when categories contributing at least 1% of fiber (e.g., tomatoes and tomato/vegetable juice, “other” vegetables, corn/peas/lima beans; carrots/sweet potatoes/winter squash; broccoli/spinach/greens) were combined, they would be the highest ranked source, providing 10.7% of fiber. 

The nutrient contribution of milk and dairy products play an important role in helping American children and adolescents meet recommendations for short-fall nutrients [24] and nutrients of public health concern [25]. In our study, milk was an important source of calcium, potassium, vitamin D, and provided many other nutrients, including vitamin A, thiamin, riboflavin, vitamins B6 and B12, phosphorus, magnesium, and zinc (Supplemental Tables). Subar *et al.* [29] found that in 1989–1991, milk (including milk drinks) and cheese provided 51.5% and 14.3% of children’s calcium intake. Sources of potassium were not reported in that study, and vitamin D food composition data were not updated or complete when that study was conducted. In our study, milk and milk drinks provided a lower percentage (38.2%), while cheese (19.4%) and other sources such as calcium-fortified fruit juice (3.2%) provided higher percentages of calcium intake than previously reported [29]. A comparison of energy sources shows milk and milk drinks contributed a lower percentage of energy in 2003–2006 than in 1989–1991, while energy contributed from less nutrient-dense foods and beverages increased. This observation is consistent with other reports [57,58,59]. Low-fat and reduced-fat milk are recommended for children after the age of 2 years. 

This study has several limitations. Food grouping can have a major influence on the ranked order of dietary sources; thus, caution is advised when comparing these data to previous reports [29,30] if there were differences in the level of aggregation (*i.e.*, the number of food groups) or disaggregation procedures used to include ingredients in food groups. Study outcomes are based on self-reported data that tend to underestimate energy intake [60]. A parent/guardian of children 2–11 y who was a proxy or assistant during the 24-h recall interview can often accurately report what the child consumed at home [61] but may not know what the child consumed outside the home [62]. A single day’s intake is not representative of an individual’s usual intake. However, the mean of the intake distribution drawn from a large, representative sample of a group is not affected by day-to-day variation [63], and since the contribution of food sources is based on mean intake data from NHANES, the use of a single 24-h recall was appropriate [64]. In general, because the food grouping in the USDA DSN database does not include ingredients of manufactured foods, disaggregated foods represent mixtures that are prepared from recipes. The USDA reduces the sodium content of mixtures if the respondent never, rarely, or occasionally uses salt in cooking, and the food was prepared at home; therefore, a large portion of the salt was added to recipes for foods prepared by restaurants, schools, and other establishments (data not shown). Finally, the updated vitamin D database that USDA recently released was appropriate for use with the 2005–2006 NHANES dietary intake data, and because vitamin D intake from foods consumed in 2003–2004 was determined using the updated food composition data, the 2003–2004 intake data may not have been representative of that time period. Any variation in food composition data affects the reliability of dietary intake estimation. It should also be noted that the major sources of vitamin D are fortified foods; changes in vitamin D fortification are regulated by a food additive rule [65]. A petition to add vitamin D to calcium-supplemented juices and fruit drinks was approved by the Food and Drug Administration and implemented in 2004 [66]. However, this change was effective only in the 2005–2006 NHANES, since the new calcium- and vitamin D-supplemented foods were not reported in the 2003–2004 NHANES.

## 5. Conclusions

This study showed that children consumed a large proportion of total energy from energy-dense, low-nutrient food groups (e.g., cake/cookies/quick bread/pastry/pie and soft drinks/soda) and identified principal sources of energy that were also major sources of nutrients (e.g., milk and milk drinks, poultry, and beef). Awareness of food and beverage sources of energy and nutrients can help health professionals design and promote effective strategies to reduce energy consumption and increase the nutrient density of the diet.

## Supplementary Files

Supplementary File 1 Supplemental Tables (PDF, 223 KB)
