# Supplementary material for: Food Sources of Energy and Nutrients among Children in the United States: National Health and Nutrition Examination Survey 2003–2006
Source: Nutrients. 2013 Jan 22;5(1):283–301. doi: 10.3390/nu5010283 (PMC3571649; doi:10.3390/nu5010283)
Supplement: Supplementary File 1 — Supplemental Tables (PDF, 223 KB) [file nutrients-05-00283-s001.pdf]

Supplemental Tables

Table S1. Food/food group sources of monounsaturated fatty acids among US children aged 2 to 18 years (from NHANES 2003–2006).

Table S2. Food/food group sources of polyunsaturated fatty acids among US children aged 2 to 18 years (from NHANES 2003–2006).

Table S3. Food/food group sources of vitamin A (retinoic acid equivalents [RAE]) among US children aged 2 to 18 years (from NHANES 2003–2006).

Table S4. Food/food group sources of vitamin E (alpha-tocoherol [AT]) among US children aged 2 to 18 years (from NHANES 2003–2006).

Table S5. Food/food group sources of vitamin C among US children aged 2 to 18 years (from NHANES 2003–2006).

Table S6. Food/food group sources of thiamin among US children aged 2 to 18 years (from NHANES 2003–2006).

Table S7. Food/Food group sources of riboflavin among US children aged 2 to 18 years (from NHANES 2003–2006).

Table S8. Food/Food group sources of niacin among US children aged 2 to 18 years (from NHANES 2003–2006).

Table S9. Food/Food group sources of vitamin B6 among US children aged 2 to 18 years (from NHANES 2003–2006).

Table S10. Food/Food group sources of folate among US children aged 2 to 18 years (from NHANES 2003–2006).

Table S11. Food/food group sources of folate (dietary folate equivalents; DFE) among US children aged 2 to 18 years (from NHANES 2003–2006).

Table S12. Food/Food group sources of vitamin B12 among US children aged 2 to 18 years (from NHANES 2003–2006).

Table S13. Food/Food group sources of phosphorus among US children aged 2 to 18 years (from NHANES 2003–2006).

Table S14. Food/Food group sources of magnesium among US children aged 2 to 18 years (from NHANES 2003–2006).

Table S15. Food/Food group sources of iron among US children aged 2 to 18 years (from NHANES 2003–2006).

Table S16. Food/Food group sources of zinc among US children aged 2 to 18 years (from NHANES 2003–2006).

Table S1. Food/food group sources of monounsaturated fatty acids among US children aged 2 to 18 years (from NHANES 2003–2006) [a].

**Food/Food Group** [b]	**Monounsaturated Fatty Acids (mean = 28.4 g)**		
	**Ranking**	**% Total**	**Cumulative %**
Cake, cookies, quick bread, pastry, pie	1	9.2	9.2
Frankfurters, sausages, luncheon meats	2	8.7	17.9
Other fats and oils	3	8.5	26.4
Beef	4	7.4	33.8
Crackers, popcorn, pretzels, chips	5	7.3	41.1
Cheese	6	7.1	48.2
Poultry	7	6.9	55.1
Milk	8	5.6	60.7
Potatoes (white)	9	5.3	66.0
Margarine and butter	10	4.5	70.5
Nuts, seeds (including butters, pastes)	11	3.8	74.3
Salad dressings, mayonnaise	12	3.6	77.9
Biscuits, corn bread, pancakes, tortillas	13	3.6	81.5
Mixtures, mostly grain	14	2.8	84.3
Pork, ham, bacon	15	2.6	86.9
Milk desserts	16	2.6	89.5
Candy, sugars and sugary foods	17	2.0	91.5

[a] Data are for children aged 2 to 18 years (n = 7332), Day 1 intakes; [b] Food groups (n = 2) contributing at least 1% in descending order: yeast breads and rolls and eggs.

Table S2. Food/food group sources of polyunsaturated fatty acids among US children aged 2 to 18 years (from NHANES 2003–2006) [a].

**Food/Food Group** [b]	**Polyunsaturated Fatty Acids (mean = 15.1 g)**		
	**Ranking**	**% Total**	**Cumulative %**
Crackers, popcorn, pretzels, chips	1	15.9	15.9
Salad dressings, mayonnaise	2	12.3	28.2
Other fats and oils	3	10.5	38.7
Cake, cookies, quick bread, pastry, pie	4	8.2	46.9
Poultry	5	8.0	54.9
Margarine and butter	6	5.3	60.2
Nuts, seeds (including butters, pastes)	7	5.3	65.5
Yeast breads and rolls	8	5.0	70.5
Biscuits, corn bread, pancakes, tortillas	9	3.7	74.2
Frankfurters, sausages, luncheon meats	10	3.6	77.8
Potatoes (white)	11	3.0	80.8
Mixtures, mostly grain	12	2.8	83.6
Milk	13	2.0	85.6

[a] Data are for children aged 2 to 18 years (n = 7332), Day 1 intakes; [b] Food groups (n = 6) contributing at least 1% in descending order: cheese; ready-to-eat cereal; eggs; candy, sugars and sugary foods; pork, ham, bacon; and beef.

Table S3. Food/food group sources of vitamin A (retinoic acid equivalents [RAE]) among US children aged 2 to 18 years (from NHANES 2003–2006) [a].

**Food/Food Group** [b]	**Vitamin A (mean = 571 µg RAE)**		
	**Ranking**	**% Total**	**Cumulative %**
Milk	1	23.3	23.3
Ready-to-eat cereal	2	15.1	38.4
Cheese	3	9.4	47.8
Carrots, sweet potatoes, winter squash	4	7.4	55.2
Margarine and butter	5	5.9	61.1
Cake, cookies, quick bread, pastry, pie	6	4.7	65.8
Milk desserts	7	4.5	70.3
Milk drinks	8	4.4	74.7
Eggs	9	3.1	77.8
Biscuits, corn bread, pancakes, tortillas	10	3.0	80.8
Mixtures, mostly grain	11	2.2	83.0

[a] Data are for children aged 2 to 18 years (n = 7332), Day 1 intakes; [b] Food groups (n = 6) contributing at least 1% in descending order: tomatoes, tomato/vegetable juice; fruit; other fats and oils; broccoli, spinach, greens; hot breakfast cereal; and poultry.

Table S4. Food/food group sources of vitamin E (alpha-tocoherol [AT]) among US children aged 2 to 18 years (from NHANES 2003–2006) [a].

**Food/Food Group** [b]	**Vitamin E (mean = 5.8 mg AT)**		
	**Ranking**	**% Total**	**Cumulative %**
Crackers, popcorn, pretzels, chips	1	14.0	14.0
Nuts, seeds (including butters, pastes)	2	9.9	23.9
Tomatoes, tomato/vegetable juice	3	9.4	33.3
Cake, cookies, quick bread, pastry, pie	4	6.4	39.7
Salad dressings, mayonnaise	5	5.8	45.5
Margarine and butter	6	5.1	50.6
Other fats and oils	7	4.5	55.1
Poultry	8	4.3	59.4
Ready-to-eat cereal	9	3.2	62.6
Mixtures, mostly grain	10	2.5	65.1
Fruit	11	2.5	67.6
Eggs	12	2.2	69.8
Fruit juice	13	2.2	72.0
Beef	14	2.1	74.1
Biscuits, corn bread, pancakes, tortillas	15	2.1	76.2
Condiments and sauces	16	2.1	78.3
Potatoes (white)	17	2.0	80.3

[a] Data are for children aged 2 to 18 years (n = 7332), Day 1 intakes; [b] Food groups (n = 9) contributing at least 1% in descending order: candy, sugars and sugary foods; milk; yeast breads and rolls; cheese; milk desserts; soup, broth, bouillon; broccoli, spinach, greens; fish and shellfish; and coffee, tea, other, nonalcoholic beverages.

Table S5. Food/food group sources of vitamin C among US children aged 2 to 18 years (from NHANES 2003–2006) [a].

Food/Food Group [b]	Vitamin C (mean = 85.7 mg)		
	**Ranking**	**% Total**	**Cumulative %**
Fruit juice	1	34.7	34.7
Fruit drinks and ades	2	23.3	58.0
Fruit	3	12.4	70.4
Ready-to-eat cereal	4	4.3	74.7
Tomatoes, tomato/vegetable juice	5	4.3	79.0
Other vegetables	6	4.2	83.2
Potatoes (white)	7	3.1	86.3
Candy, sugars and sugary foods	8	3.1	89.4
Broccoli, spinach, greens	9	2.7	92.1

[a] Data are for children aged 2 to 18 years (*n* = 7332), Day 1 intakes; [b] Food groups (*n* = 2) contributing at least 1% in descending order: condiments and sauces and crackers, popcorn, pretzels, chips.

Table S6. Food/food group sources of thiamin among US children aged 2 to 18 years (from NHANES 2003–2006) [a].

Food/Food Group [b]	Thiamin (mean = 1.6 mg)		
	**Ranking**	**% Total**	**Cumulative %**
Ready-to-eat cereal	1	17.1	17.1
Yeast breads and rolls	2	13.5	30.6
Milk	3	7.0	37.6
Flour, bran, baking ingredients	4	6.6	44.2
Biscuits, corn bread, pancakes, tortillas	5	5.5	49.7
Pork, ham, bacon	6	5.5	55.2
Cake, cookies, quick bread, pastry, pie	7	5.2	60.4
Pasta	8	5.0	65.4
Mixtures, mostly grain	9	3.8	69.2
Fruit juice	10	3.1	72.3
Crackers, popcorn, pretzels, chips	11	3.0	75.3
Potatoes (white)	12	2.9	78.2
Frankfurters, sausages, luncheon meats	13	2.5	80.7

[a] Data are for children aged 2 to 18 years (*n* = 7332), Day 1 intakes; [b] Food groups (*n* = 8) contributing at least 1% in descending order: poultry; rice, cooked grains; fruit; milk drinks; fruit drinks and ades; soup, broth, bouillon; beef; and legumes.

Table S7. Food/Food group sources of riboflavin among US children aged 2 to 18 years (from NHANES 2003–2006) [a].

Food/Food Group [b]	Riboflavin (mean = 2.2 mg)		
	**Ranking**	**% Total**	**Cumulative %**
Milk	1	23.9	23.9
Ready-to-eat cereal	2	13.6	37.5
Yeast breads and rolls	3	7.2	44.7
Cheese	4	5.3	50.0
Cake, cookies, quick bread, pastry, pie	5	3.9	53.9
Milk drinks	6	3.8	57.7
Biscuits, corn bread, pancakes, tortillas	7	3.5	61.2
Flour, bran, baking ingredients	8	3.5	64.7
Poultry	9	3.0	67.7
Milk desserts	10	2.9	70.6
Eggs	11	2.7	73.3
Crackers, popcorn, pretzels, chips	12	2.6	75.9
Beef	13	2.6	78.5
Mixtures, mostly grain	14	2.4	80.9

[a] Data are for children aged 2 to 18 years (*n* = 7332), Day 1 intakes; [b] Food groups (*n* = 7) contributing at least 1% in descending order: frankfurters, sausages, luncheon meats; pasta; pork, ham, bacon; fruit drinks and ades; fruit juice; fruit; and condiments and sauces.

Table S8. Food/Food group sources of niacin among US children aged 2 to 18 years (from NHANES 2003–2006) [a].

Food/Food Group [b]	Niacin (mean = 21.4 mg)		
	**Ranking**	**% Total**	**Cumulative %**
Ready-to-eat cereal	1	16.2	16.2
Poultry	2	13.8	30.0
Yeast breads and rolls	3	10.1	40.1
Beef	4	7.5	47.6
Flour, bran, baking ingredients	5	4.2	51.8
Crackers, popcorn, pretzels, chips	6	4.0	55.8
Cake, cookies, quick bread, pastry, pie	7	4.0	59.8
Biscuits, corn bread, pancakes, tortillas	8	3.9	63.7
Frankfurters, sausages, luncheon meats	9	3.8	67.5
Pork, ham, bacon	10	3.2	70.7
Potatoes (white)	11	3.0	73.7
Mixtures, mostly grain	12	2.9	76.6
Tomatoes, tomato/vegetable juice	13	2.7	79.3
Nuts, seeds (including butters, pastes)	14	2.5	81.8
Pasta	15	2.5	84.3

[a] Data are for children aged 2 to 18 years (*n* = 7332), Day 1 intakes; [b] Food groups (*n* = 7) contributing at least 1% in descending order: fish and shellfish; fruit drinks and ades; milk; rice, cooked grains; fruit juice; soup, broth, bouillon; and fruit.

Table S9. Food/Food group sources of vitamin B6 among US children aged 2 to 18 years (from NHANES 2003–2006) [a].

Food/Food Group [b]	Vitamin B6 (mean = 1.7 mg)		
	Ranking	% Total	Cumulative %
Ready-to-eat cereal	1	22.3	22.3
Poultry	2	7.6	29.9
Beef	3	6.8	36.7
Milk	4	6.1	42.8
Potatoes (white)	5	6.1	48.9
Fruit	6	4.3	53.2
Fruit juice	7	4.2	57.4
Crackers, popcorn, pretzels, chips	8	3.6	61.0
Biscuits, corn bread, pancakes, tortillas	9	3.2	64.2
Pork, ham, bacon	10	2.8	67.0
Cake, cookies, quick bread, pastry, pie	11	2.7	69.7
Frankfurters, sausages, luncheon meats	12	2.6	72.3
Tomatoes, tomato/vegetable juice	13	2.5	74.8
Fruit drinks and ades	14	2.3	77.1
Yeast breads and rolls	15	2.3	79.4

[a] Data are for children aged 2 to 18 years (*n* = 7332), Day 1 intakes; [b] Food groups (*n* = 8) contributing at least 1% in descending order: other vegetables; nuts, seeds (including butters, pastes); milk drinks; mixtures, mostly grain; cheese; coffee, tea, other, nonalcoholic beverages; eggs; and condiments and sauces.

Table S10. Food/Food group sources of folate among US children aged 2 to 18 years (from NHANES 2003–2006) [a].

Food/Food Group [b]	Folate (mean = 387 μg)		
	Ranking	% Total	Cumulative %
Ready-to-eat cereal	1	25.7	25.7
Yeast breads and rolls	2	14.0	39.7
Flour, bran, baking ingredients	3	6.0	45.7
Pasta	4	5.5	51.2
Cake, cookies, quick bread, pastry, pie	5	5.1	56.3
Biscuits, corn bread, pancakes, tortillas	6	4.6	60.9
Crackers, popcorn, pretzels, chips	7	4.0	64.9
Mixtures, mostly grain	8	3.8	68.7
Milk	9	3.7	72.4
Fruit juice	10	3.4	75.8
Rice, cooked grains	11	2.5	78.3
Legumes	12	2.1	80.4

[a] Data are for children aged 2 to 18 years (*n* = 7332), Day 1 intakes; [b] Food groups (*n* = 7) contributing at least 1% in descending order: fruit; potatoes (white); nuts, seeds (including butters, pastes); eggs; other vegetables; broccoli, spinach, greens; and tomatoes and tomato/vegetable juice.

Table S11. Food/food group sources of folate (dietary folate equivalents; DFE) among US children aged 2 to 18 years (from NHANES 2003–2006) [a].

Food/Food Group [b]	Folate (mean = 548 µg DFE)		
	**Ranking**	**% Total**	**Cumulative %**
Ready-to-eat cereal	1	30.3	30.3
Yeast breads and rolls	2	14.8	45.1
Flour, bran, baking ingredients	3	6.5	51.6
Pasta	4	6.3	57.9
Cake, cookies, quick bread, pastry, pie	5	5.4	63.3
Biscuits, corn bread, pancakes, tortillas	6	5.0	68.3
Mixtures, mostly grain	7	3.9	72.2
Crackers, popcorn, pretzels, chips	8	3.7	75.9
Rice, cooked grains	9	3.0	78.9
Milk	10	2.6	81.5
Fruit juice	11	2.4	83.9

[a] Data are for children aged 2 to 18 years (*n* = 7332), Day 1 intakes; [b] Food groups (*n* = 3) contributing at least 1% in descending order: legumes; fruit; and potatoes (white).

Table S12. Food/Food group sources of vitamin B12 among US children aged 2 to 18 years (from NHANES 2003–2006) [a].

Food/Food Group [b]	Vitamin B12 (mean = 5.3 µg)		
	**Ranking**	**% Total**	**Cumulative %**
Milk	1	24.7	24.7
Ready-to-eat cereal	2	18.5	43.2
Beef	3	14.7	57.9
Cheese	4	6.4	64.3
Fish and shellfish	5	5.2	69.5
Frankfurters, sausages, luncheon meats	6	4.7	74.2
Mixtures, mostly grain	7	3.2	77.4
Milk drinks	8	2.9	80.3
Eggs	9	2.7	83.0
Fruit drinks and ades	10	2.3	85.3
Poultry	11	2.2	87.5
Milk desserts	12	2.1	89.6

[a] Data are for children aged 2 to 18 years (*n* = 7332), Day 1 intakes; [b] Food groups (*n* = 5) contributing at least 1% in descending order: pork, ham, bacon; biscuits, corn bread, pancakes, tortillas; cake, cookies, quick bread, pastry, pie; yogurt and organ meats.

Table S13. Food/Food group sources of phosphorus among US children aged 2 to 18 years (from NHANES 2003–2006) [a].

**Food/Food Group** [b]	**Phosphorus (mean = 1265 mg)**		
	**Ranking**	**% Total**	**Cumulative %**
Milk	1	21.3	21.3
Cheese	2	11.8	33.1
Poultry	3	6.6	39.7
Beef	4	5.0	44.7
Biscuits, corn bread, pancakes, tortillas	5	4.6	49.3
Yeast breads and rolls	6	3.9	53.2
Crackers, popcorn, pretzels, chips	7	3.4	56.6
Cake, cookies, quick bread, pastry, pie	8	3.3	59.9
Milk drinks	9	3.3	63.2
Mixtures, mostly grain	10	3.1	66.3
Frankfurters, sausages, luncheon meats	11	2.8	69.1
Ready-to-eat cereal	12	2.6	71.7
Pork, ham, bacon	13	2.6	74.3
Milk desserts	14	2.5	76.8
Potatoes (white)	15	2.4	79.2

[a] Data are for children aged 2 to 18 years (n = 7332), Day 1 intakes; [b] Food groups (n = 10) contributing at least 1% in descending order: eggs; nuts, seeds (including butters, pastes); flour, bran, baking ingredients; pasta; soft drinks, soda (including diet); fish and shellfish; legumes; candy, sugars and sugary foods; fruit juice; and yogurt.

Table S14. Food/Food group sources of magnesium among US children aged 2 to 18 years (from NHANES 2003–2006) [a].

**Food/Food Group** [b]	**Magnesium (mean = 231 mg)**		
	**Ranking**	**% Total**	**Cumulative %**
Milk	1	13.3	13.3
Crackers, popcorn, pretzels, chips	2	7.3	20.6
Yeast breads and rolls	3	5.9	26.5
Ready-to-eat cereal	4	4.2	30.7
Cake, cookies, quick bread, pastry, pie	5	4.2	34.9
Fruit juice	6	4.1	39.0
Poultry	7	4.1	43.1
Potatoes (white)	8	3.9	47.0
Nuts, seeds (including butters, pastes)	9	3.8	50.8
Cheese	10	3.5	54.3
Fruit	11	3.4	57.7
Biscuits, corn bread, pancakes, tortillas	12	3.0	60.7
Beef	13	2.9	63.6
Milk drinks	14	2.8	66.4
Coffee, tea, other, nonalcoholic beverages	15	2.7	69.1
Tomatoes, tomato/vegetable juice	16	2.7	71.8
Pasta	17	2.5	74.3
Candy, sugars and sugary foods	18	2.3	76.6
Mixtures, mostly grain	19	2.2	78.8
Milk desserts	20	2.2	81.0
Legumes	21	2.1	83.1

[a] Data are for children aged 2 to 18 years (n = 7332), Day 1 intakes; [b] Food groups (n = 8) contributing at least 1% in descending order: flour, bran, baking ingredients; frankfurters, sausages, luncheon meats; fruit drinks and ades; pork, ham, bacon; condiments and sauces; other vegetables; fish and shellfish; and rice, cooked grains.

Table S15. Food/Food group sources of iron among US children aged 2 to 18 years (from NHANES 2003–2006) [a].

**Food/Food Group** [b]	**Iron (mean = 15.2 mg)**		
	**Ranking**	**% Total**	**Cumulative %**
Ready-to-eat cereal	1	22.9	22.9
Yeast breads and rolls	2	11.9	34.8
Cakes, cookies, quick bread, pastry, pie	3	6.3	41.1
Flour, bran, baking ingredients	4	5.2	46.3
Beef	5	5.1	51.4
Biscuits, corn bread, pancakes, tortillas	6	4.9	56.3
Crackers, popcorn, pretzels, chips	7	4.3	60.6
Mixtures, mostly grain	8	3.1	63.7
Fruit juice	9	2.9	66.6
Poultry	10	2.7	69.3
Pasta	11	2.7	72.0
Tomatoes, tomato/vegetable juice	12	2.4	74.4
Frankfurters, sausages, luncheon meats	13	2.0	76.4
Soft drinks, soda (includes diet)	14	2.0	78.4

[a] Data are for children aged 2 to 18 years (n = 7332), Day 1 intakes; [b] Food groups (n = 9) contributing at least 1% in descending order: potatoes (white); legumes; eggs; rice, cooked grains; candy, sugars and sugary foods; hot breakfast cereal; soup, broth, bouillon; fruit; and milk drinks.

Table S16. Food/Food group sources of zinc among US children aged 2 to 18 years (from NHANES 2003–2006) [a].

**Food/Food Group** [b]	**Zinc (mean = 11.2 mg)**		
	**Ranking**	**% Total**	**Cumulative %**
Beef	1	16.6	16.6
Ready-to-eat cereal	2	14.3	30.9
Milk	3	10.8	41.7
Cheese	4	8.0	49.7
Poultry	5	5.4	55.1
Frankfurters, sausages, luncheon meats	6	4.4	59.5
Yeast breads and rolls	7	4.0	63.5
Crackers, popcorn, pretzels, chips	8	3.7	67.2
Mixture, mainly grain	9	3.1	70.3
Pork, ham, bacon	10	2.7	73.0
Cake, cookies, quick bread, pastry, pie	11	2.3	75.3

[a] Data are for children aged 2 to 18 years (n = 7332), Day 1 intakes; [b] Food groups (n = 12) contributing at least 1% in descending order: milk drinks; milk desserts; legumes; nuts, seeds (including butters, pastes); candy, sugar and sugary foods; pasta; biscuits, corn bread, pancakes, tortillas; potatoes (white); flour, bran, baking ingredients; eggs; fish and shellfish; and tomatoes, tomato/vegetable juice.
